# A novel DNA methylation-based model that effectively predicts prognosis in hepatocellular carcinoma

**DOI:** 10.1042/BSR20203945

**Published:** 2021-03-10

**Authors:** Xiang-Yong Hao, An-Qiang Li, Hao Shi, Tian-Kang Guo, Yan-Fei Shen, Yuan Deng, Li-Tian Wang, Tao Wang, Hui Cai

**Affiliations:** 1 Department of General Surgery, Gansu Provincial Hospital, Lanzhou, China; 2 Key Laboratory of Molecular Diagnosis and Precision Treatment of Surgical Tumors in Gansu Province, Lanzhou, China; 3 Department of Medical Management, Gansu Provincial Hospital, Lanzhou, China

**Keywords:** DNA methylation, GEO, hepatocellular carcinoma, prognosis, TCGA

## Abstract

**Purpose:** To build a novel predictive model for hepatocellular carcinoma (HCC) patients based on DNA methylation data.

**Methods:** Four independent DNA methylation datasets for HCC were used to screen for common differentially methylated genes (CDMGs). Gene Ontology (GO) enrichment, and Kyoto Encyclopedia of Genes and Genomes (KEGG) pathway enrichment analysis were used to explore the biological roles of CDMGs in HCC. Univariate Cox analysis and least absolute shrinkage and selection operator (LASSO) Cox analysis were performed to identify survival-related CDMGs (SR-CDMGs) and to build a predictive model. The importance of this model was assessed using Cox regression analysis, propensity score-matched (PSM) analysis and stratification analysis. A validation group from the Cancer Genome Atlas (TCGA) was constructed to further validate the model.

**Results:** Four SR-CDMGs were identified and used to build the predictive model. The risk score of this model was calculated as follows: risk score = (0.01489826 × methylation level of *WDR69*) + (0.15868618 × methylation level of *HOXB4*) + (0.16674959 × methylation level of *CDKL2*) + (0.16689301 × methylation level of *HOXA10*). Kaplan–Meier analysis demonstrated that patients in the low-risk group had a significantly longer overall survival (OS; log-rank *P*-value =0.00071). The Cox model multivariate analysis and PSM analysis identified the risk score as an independent prognostic factor (*P*<0.05). Stratified analysis results further confirmed this model performed well. By analyzing the validation group, the results of receiver operating characteristic (ROC) curve analysis and survival analysis further validated this model.

**Conclusion:** Our DNA methylation-based prognosis predictive model is effective and reliable in predicting prognosis for patients with HCC.

## Introduction

Hepatocellular carcinoma (HCC) is one of the most prevalent malignant tumors worldwide, with its incidence increasing over the last 10 years [1]. Current TNM staging systems and Barcelona Clinic Liver Cancer staging systems were constructed based on the anatomical features of the tumor and clinic-pathological information [2,3]. These approaches remain the most commonly used staging systems for patients with HCC. Therefore, the prognoses of patients with HCC vary significantly even among those with similar clinical staging. Therefore, more prognostic and survival-related factors of patients with HCC must be identified and applied to improve prognostic accuracy.

DNA methylation is an important epigenetic modification that is involved in the regulation of gene expression and genome stability [4]. There is mounting evidence that aberrant DNA methylation in promoter regions contributes to the carcinogenesis, progression and prognosis of many tumors [5–13]. For example, promoter methylation of *P16* (*CDKN2A* or *Ink4a*) may directly inactivate gene transcription and drive cancer metastasis [11]. Besides, DNA methylation in the promoter regions of *IRAK3* is clearly associated with the tumor stage and clinical outcomes of HCC patients [10]. Moreover, the development of next-generation sequencing and microarrays further improves our understanding of DNA methylation associated with tumors.

Here, we aimed to identify important prognosis-related methylation signatures. Furthermore, we constructed a composite predictive model for HCC patients using CpG methylation data.

## Materials and methods

### Retrieval of DNA methylation data

Four independent DNA methylation datasets for HCC examined by HumanMethylation450 Bead Chip (Illumina) were downloaded from public databases. Three of the datasets (GSE54503, GSE77269 and GSE56588 datasets) were obtained from the Gene Expression Omnibus database (https://www.ncbi.nlm.nih.gov/geo/), while the fourth dataset (from the Cancer Genome Atlas [TCGA], https://cancergenome.nih.gov/) contained corresponding clinical prognostic information. Each dataset contained information on more than ten cancer tissues and ten adjacent non-cancerous tissues. For the TCGA dataset, non-HCCs and patients without DNA methylation information were removed; 365 HCC samples and 50 non-tumor samples were retained. The methylation levels of each probe are expressed as a β-value.

### Identification of common differentially methylated genes

In the four datasets, we used the Chip Analysis Methylation Pipeline Bio-conductor package to identify differentially methylated genes (DMGs) between HCC and adjacent non-cancerous tissues [14]. Only DNA methylation at CpG islands within the promoter region of the gene was screened for analysis. The promoter region indicates loci residing within the Tss1500 (200–1500 bps upstream of the transcription start site) and Tss200 (200 bps upstream of the transcription start site) of known genes [15]. DNA methylation sites on sex chromosomes, probes containing single nucleotide polymorphisms as reported by Zhou et al. [16] and probes aligning to multiple locations as identified by Nordlund et al. [17] were removed. In addition, probes with β values in less than 95% of samples were removed. Then, NAs were replaced by the k-nearest neighbor imputation procedure [18,19]. Only probes with adjusted *P*<0.05 and a |log(fold change)| ≥ 1 were regarded as differentially methylated probes (DMPs). The genes mapped by DMPs were confirmed as DMGs. DMGs that were common across the four datasets (common differentially methylated genes, CDMGs) were selected for further analysis.

### Bioinformatics analysis for CDMGs

CDMGs, including common hyper-methylated genes and common hypo-methylated genes obtained from the four independent datasets, were subjected to Gene Ontology (GO) enrichment and Kyoto Encyclopedia of Genes and Genomes (KEGG) pathway enrichment analysis via DAVID 6.8 (https://david.ncifcrf.gov/) to investigate their activities in HCC [20,21]. The STRING database (https://string-db.org/) and Cytoscape 3.6.0 were used to build a protein–protein interaction (PPI) network of CDMGs. The STRING database is a precomputed resource used to investigate protein regulatory networks in different diseases [22–24]. The PPI score (confidence) was set as 0.4.

### Identification of survival-related CDMGs and construction of the predictive model

After removing patients with detectable residual tumors (R1 and R2) and patients who received adjuvant treatment (including radiation therapy, chemotherapy and embolization treatment), 296 HCC samples in the TCGA dataset were retained to screen for survival-related methylated markers and to construct a predictive model. Univariate Cox regression analysis was used to identify risk markers among the CDMGs that were clearly associated with the overall survival (OS) of HCC. The results were considered significant when *P*<0.05. The risk markers from univariate Cox analysis were further analyzed using the least absolute shrinkage and selection operator (LASSO) Cox regression model for survival-related CDMG (SR-CDMG) selection and classifier construction. LASSO Cox analysis is a regularized Cox regression approach that is suitable for analyzing high-dimensional data of small sample size [25]. In this way, it was possible to select a subset of variables that best predict OS while reducing the overfitting of data [26]. Ten-fold cross-validation with minimum criteria was applied to identify the optimal parameter λ in the LASSO Cox model [27,28]. Using this model, the regression coefficients of most features were shrunk towards zero, allowing the most useful predictors (representing SR-CDMGs) to be identified. Then, the SR-CDMGs with non-zero coefficient were used to build a predictive model for HCC. A risk score for each patient was computed according to the summation of the methylation levels (β value) multiplied by the corresponding coefficients from the LASSO Cox analysis.

We divided patients into two groups according to the median values of SR-CDMGs and the risk score. Kaplan–Meier survival curves were used to analyze the correlation of SR-CDMGs and the risk score with OS. In addition, univariate and multivariate-adjusted Cox regression analysis, propensity score-matched (PSM) analysis and stratification analysis were performed to estimate the importance of the predictive model in the prognosis of HCC.

Moreover, in order to further validate the predictive model that we constructed, HCC samples with DNA methylation information and clinical prognostic information in the TCGA dataset were randomly selected to construct a validation group. Then the predictive model was verified by receiver operating characteristic (ROC) curve and survival analysis. Area under the ROC curve (AUC) values for 1-, 3-, and 5-year survival of the predictive model were also calculated.

### Statistical analysis

R software 3.3.4 (https://www.r-project.org/) was used to perform the statistical analysis and graphic visualization. The results were considered statistically significant at a two-tailed *P* value <0.05. Differences in continuous variables between the two groups were analyzed using the non-parametric Mann–Whitney U test or Student’s *t* test [29]. DNA methylation data from HumanMethylation450 Bead Chip were processed with the R package ‘ChAMP’ [14]. LASSO Cox analysis was performed with the R package ‘glmnet’ [30,31]. Cox regression analysis was carried out with the package ‘survival’ [32]. The R package ‘MatchIt’ was used for PSM analysis [33]. The ROC curve analyses were performed using the R package pROC [34].

## Results

### Identification of CDMGs and bioinformatics analysis

**Figure 1:**
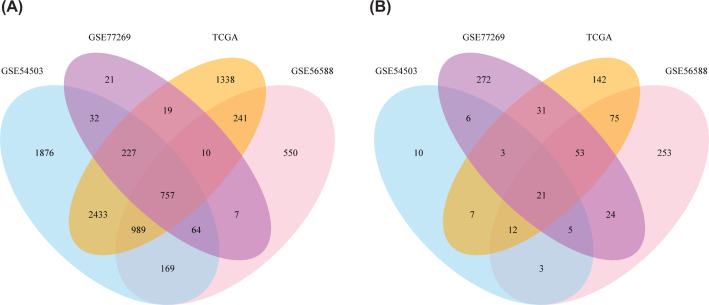
CDMGs in four datasets (**A**) Venn diagram showing 757 common hyper-methylated genes in HCC samples. (**B**) Venn diagram showing 21 common hypo-methylated genes in HCC samples.

To improve the reliability of our results, four independent datasets were used to recognize differentially methylated markers of tumor vs adjacent non-cancerous tissues. A total of 778 CDMGs (Additional file 1: Supplementary Table S1) from the four datasets were regarded as credible, including 757 hyper-methylated genes (Figure 1A) and 21 hypo-methylated genes (Figure 1B) in HCC.

**Figure 2:**
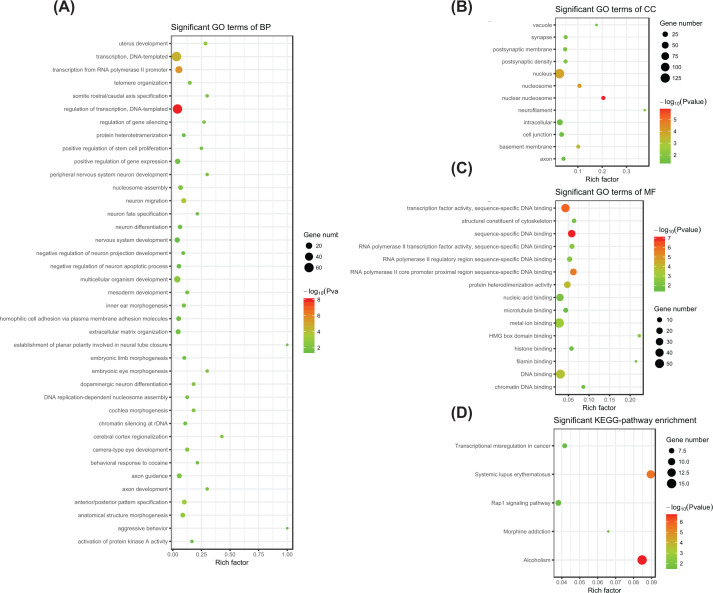
GO enrichment analysis and KEGG pathway enrichment analysis of CDMGs (**A**) Significant GO terms for biological processes. (**B**) Significant GO terms for cellular components. (**C**) Significant GO terms for molecular functions. (**D**) Significant KEGG pathways. Terms and pathways with *P*<0.05 were here considered significant.

**Figure 3:**
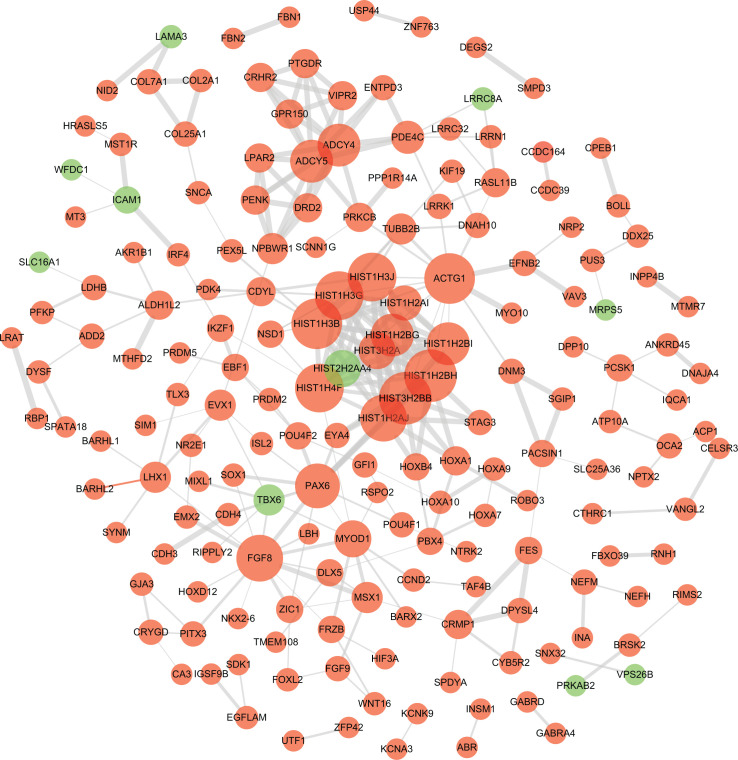
PPI network of CDMGs After removing the unconnected nodes, 180 nodes and 299 edges were retained. Red nodes represent common hyper-methylated genes, and blue nodes represent common hypo-methylated genes. Node size is proportional to the node’s degree. Line thickness indicates the strength of data support. The PPI score was set at 0.4.

To explore the biological roles of the 778 CDMGs, GO enrichment analysis and KEGG pathway analysis were performed. Significant GO terms and pathways (*P*<0.05) are shown in Figure 2. The main terms of the biological processes include the regulation of tissue development, morphogenesis and transcription (Figure 2A). Terms related to the synapse and nucleosome were significantly enriched in the cellular component (Figure 2B). The main terms related to molecular functions were clearly related to DNA binding (Figure 2C). In addition, pathways involving transcriptional misregulation in cancer, Rap1 signaling, systemic lupus erythematosus, morphine addiction and alcoholism were enriched in KEGG analysis (Figure 2D). To evaluate the interactive relationships among all CDMGs and to elucidate the molecular mechanisms underlying HCC, a PPI network with 180 nodes and 299 edges was constructed (Figure 3).

### Identification of SR-CDMGs and construction of the predictive model

**Figure 4:**
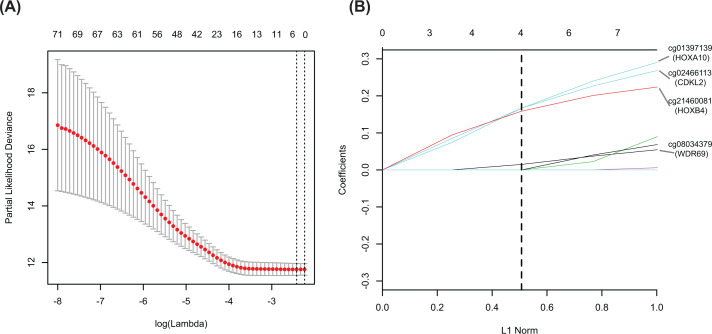
Four SR-CDMGs selected through LASSO Cox analysis (**A**) Ten-fold cross-validation for selection of the parameter λ. The solid vertical lines represent partial likelihood deviance ± standard error (SE). The two dotted vertical lines were drawn at the optimal values using minimum criteria (right) and 1-SE criteria (left). The parameter λ = 0.08905296 (log(λ) = −2.418524) was chosen using minimum criteria. (**B**) LASSO coefficients of the four SR-CDMGs. The dotted vertical line was drawn at the λ value chosen using minimum criteria. The upper axis represents the number of non-zero coefficients at each λ (the degrees of freedom for the LASSO model). The L1 norm represents the summation of absolute non-zero coefficients at each λ. The vertical axis represents the values of non-zero coefficients at each λ. The LASSO coefficients of the four SR-CDMGs *HOXA10, CDKL2, HOXB4* and *WDR69* were 0.16689301, 0.16674959, 0.15868618 and 0.01489826, respectively.

**Figure 5:**
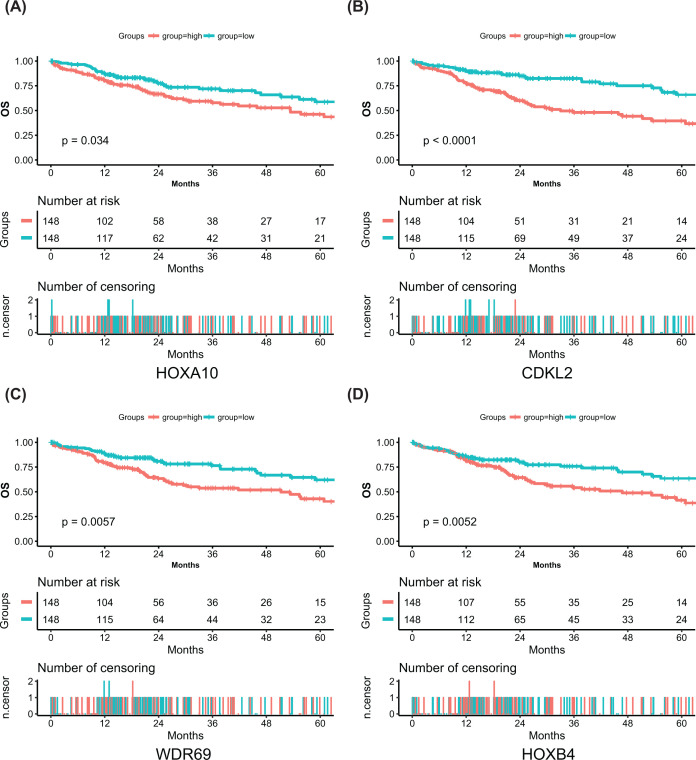
Kaplan–Meier survival analysis for the four SR-CDMGs (**A**) Survival curve of *HOXA10*. (**B**) Survival curve of *CDKL2*. (**C**) Survival curve of *WDR69*. (**D**) Survival curve of *HOXB4*. Two-hundred and ninety-six patients from the TCGA were divided into a hyper-methylated and hypo-methylated groups according to the median value of the four SR-CDMGs. *P*-values were calculated with the log-rank test.

For the 778 candidate CDMGs, univariate Cox regression analysis was conducted to screen significant risk markers associated with OS in the TCGA dataset. Seventy-two CDMGs with *P*<0.05 were identified (Additional file 2: Supplementary Table S2). To identify SR-CDMGs among these 72 CDMGs, LASSO Cox analysis was performed. The parameter log(λ) = −2.418524 (λ = 0.08905296), chosen by the ten-fold cross-validation method with minimum criteria, produced the best value (Figure 4A). Four genes with non-zero coefficients were identified as SR-CDMGs (*HOXA10, CDKL2, HOXB4* and *WDR69*) (Figure 4B); all four genes had higher methylation levels in HCC samples (Additional file 3: Supplementary Figure S1A–D). Based on the median values of the methylation levels (β values) of these four SR-CDMGs, patients in the TCGA dataset were divided into hyper-methylated and hypo-methylated groups. All SR-CDMGs were closely related to poor OS (log-rank *P*-value <0.05) (Figure 5A–D).

**Figure 6:**
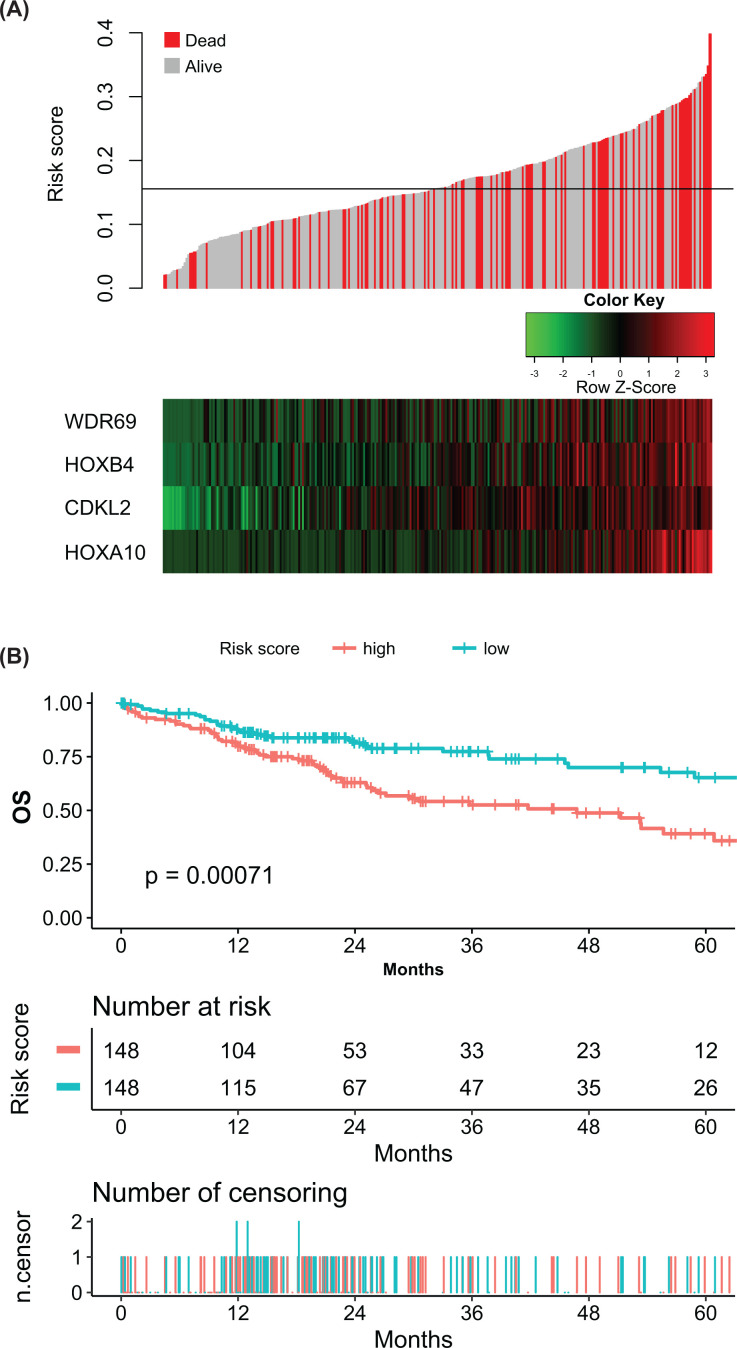
Survival analysis of the predictive model (**A**) Distribution of the risk score of the predictive model, the survival status of patients and the methylation level of the four SR-CDMGs. (**B**) Kaplan–Meier survival curve of the predictive model. Patients were divided into low-risk and high-risk groups according to the median value of the risk score. *P*-values were calculated with the log-rank test.

To build an individualized predictive model for HCC based on the four SR-CDMGs, a risk score for each patient in the TCGA dataset was computed by summing the methylation levels multiplied by the corresponding coefficients from the LASSO Cox analysis: risk score = (0.01489826 × methylation level of *WDR69*) + (0.15868618 × methylation level of *HOXB4*) + (0.16674959 × methylation level of *CDKL2*) + (0.16689301 × methylation level of *HOXA10*). The distributions of the risk score, survival status and the methylation level of the four SR-CDMGs are presented in Figure 6A. Patients with higher risk scores generally had lower survival rates than with patients with low risk scores. Patients were divided into low-risk and high-risk groups according to the median value of the risk score. Kaplan–Meier analysis demonstrated that patients in the low-risk group had a significantly longer OS than those in the high-risk group (log-rank *P*-value = 0.00071) (Figure 6B).

### Cox regression analysis and PSM analysis of the predictive model

Univariate Cox analysis and Cox model multivariate analysis were used to examine the prognostic factors for OS in HCC patients (Table 1). The unadjusted univariate Cox model showed that (i) the risk score (*P*<0.001), (ii) the American Joint Committee on Cancer (AJCC) stage (*P*=0.008, II, III and IV vs I) and (iii) α-fetoprotein (AFP) levels (*P*=0.015) were statistically related to the prognosis of HCC. A multivariate Cox model adjusted for important clinical factors (such as sex, age and race) showed that the risk score remained a significant independent risk factor for OS (*P*<0.05).

**Table 1:** Cox regression analysis of risk factors associated with OS in TCGA

	Unadjusted		Adjusted 1		Adjusted 2	
Variables	HR (95% CI)	*P*-value	HR (95% CI)	*P*-value	HR (95% CI)	*P*-value
Risk score						
Low						
High	1.997 (1.328–3.003)	**<0.001**	2.400 (1.312–4.390)	**0.005**	2.54 (1.485–4.347)	**<0.001**
Sex						
Female						
Male	0.803 (0.537–1.199)	0.283	1.072 (0.535–2.148)	0.845		
Age (years)						
≤60						
>60	1.200 (0.810–1.780)	0.363	1.359 (0.717–2.577)	0.348		
Race						
Other^*^						
White	1.363 (0.903–2.057)	0.141	1.876 (0.925–3.801)	0.081		
Risk factors^†^						
No						
Yes	0.666 (0.435–1.019)	0.061	1.063 (0.523–2.159)	0.866		
Grade						
G1 and G2						
G3 and G4	1.140 (0.762–1.704)	0.525	1.298 (0.697–2.416)	0.411		
AJCC						
I						
II and III and IV	1.775 (1.165–2.706)	**0.008**	1.088 (0.555–2.133)	0.806	1.261 (0.772–2.061)	0.354
Vascular invasion						
No						
Yes	1.405 (0.884–2.233)	0.150	1.168 (0.566–2.408)	0.674		
AFP (ng/ml)						
<20						
≧20	1.890 (1.163–3.073)	**0.010**	2.020 (1.075–3.798)	**0.029**	1.551 (0.937–2.567)	0.088
History of other malignancy						
No						
Yes	1.232 (0.620–2.449)	0.552	1.863 (0.670–5.185)	0.233		
Family history of cancer						
No						
Yes	1.114 (0.732–1.696)	0.615	0.897 (0.483–1.665)	0.730		

Adjusted 1: Adjusted covariates include all the indicators above.

Adjusted 2: Adjusted covariates include the prognostic factors from unadjusted univariate COX analysis.

* Represents Asian and black patients.

† Represents patients with the history of hepatitis B, hepatitis C, hemochromatosis, cirrhosis, alcohol consumption, non-alcoholic fatty liver disease or α-1 antitrypsin deficiency.

The values in bold denote that the difference between the two groups is statistically significant (*P*<0.05).

To reduce the confounding effects of the outcomes of the statistical comparisons between high- and low-risk score groups, PSM was performed to balance all known variables. The baseline characteristics of the two groups are shown in Table 2. Univariate Cox and multivariate-adjusted Cox regression analyses after PSM support the result that the risk score was an independent risk factor associated with poor prognosis (Table 3, all *P*-values of the risk scores were under 0.05).

**Table 2:** Baseline characteristics of the patients in the PSM cohort stratified by risk score (high vs low group)

	PSM cohort		
Variable	Risk score: High (*n*=89)	Risk score: Low (*n*=89)	*P*
Age (years), mean (SD)	60.31 (12.08)	58.42 (11.67)	0.288
Sex			0.263
Male	56 (62.9)	64 (71.9)	
Female	33 (37.1)	25 (28.1)	
Race			0.540
White	38 (42.7)	33 (37.1)	
Other	51 (57.3)	56 (62.9)	
History of other malignancy			1.000
Yes	7 (7.9)	7 (7.9)	
No	82 (92.1)	82 (92.1)	
Family history of cancer			0.108
Yes	34 (38.2)	23 (25.8)	
No	55 (61.8)	66 (74.2)	
Risk factors			0.112
Yes	63 (70.8)	73 (82.0)	
No	26 (29.2)	16 (18.0)	
Grade			0.880
G1 and G2	49 (55.1)	47 (52.8)	
G3 and G4	40 (44.9)	42 (47.2)	
AJCC			0.759
I	52 (58.4)	55 (61.8)	
II and III and IV	37 (41.6)	34 (38.2)	
Vascular invasion			0.339
Yes	33 (37.1)	26 (29.2)	
No	56 (62.9)	63 (70.8)	
AFP (ng/ml)			0.450
≥20	42 (47.2)	36 (40.4)	
<20	47 (52.8)	53 (59.6)	

**Table 3:** Cox regression analysis of risk factors associated with OS in TCGA after PSM analysis

	Unadjusted		Adjusted 1		Adjusted 2	
Variables	HR (95% CI)	*P*-value	HR (95% CI)	*P*-value	HR (95% CI)	*P*-value
Risk Score						
Low						
High	2.815 (1.554–5.097)	**<0.001**	2.536 (1.355–4.749)	**0.004**	2.481 (1.364–4.513)	**0.003**
Sex						
Female						
Male	0.576 (0.329–1.01)	0.054	1.102 (0.546–2.222)	0.787		
Age (years)						
≤60						
>60	1.567 (0.895–2.744)	0.116	1.37 (0.712–2.634)	0.346		
Race						
Other^*^						
White	2.168 (1.222–3.847)	**0.008**	2.049 (0.994–4.223)	0.052	2.056 (1.153–3.667)	**0.015**
Risk factors^†^						
No						
Yes	0.742 (0.396–1.393)	0.353	1.133 (0.547–2.349)	0.736		
Grade						
G1 and G2						
G3 and G4	1.264 (0.726–2.198)	0.408	1.248 (0.663–2.351)	0.492		
AJCC						
I						
II and III and IV	1.18 (0.674–2.066)	0.561	0.961 (0.475–1.943)	0.912		
Vascular invasion						
No						
Yes	1.411 (0.775–2.569)	0.26	1.283 (0.607–2.711)	0.514		
AFP (ng/ml)						
<20						
≧20	2.015 (1.146–3.542)	**0.015**	2.047 (1.079–3.886)	**0.028**	1.954 (1.105–3.457)	**0.021**
History of other malignancy						
No						
Yes	1.944 (0.76–4.97)	0.165	2.053 (0.725–5.814)	0.176		
Family history of cancer						
No						
Yes	1.307 (0.746–2.291)	0.349	0.846 (0.451–1.587)	0.602		

Adjusted 1: Adjusted covariates include all the indicators above.

Adjusted 2: Adjusted covariates include the prognostic factors from unadjusted univariate COX analysis.

* Represents Asian and black patients.

† Represents patients with the history of hepatitis B, hepatitis C, hemochromatosis, cirrhosis, alcohol consumption, non-alcoholic fatty liver disease or α-1 antitrypsin deficiency.

The values in bold format denote that the difference between the two groups is statistically significant (*P*<0.05).

### Stratification analysis of the predictive model

**Figure 7:**
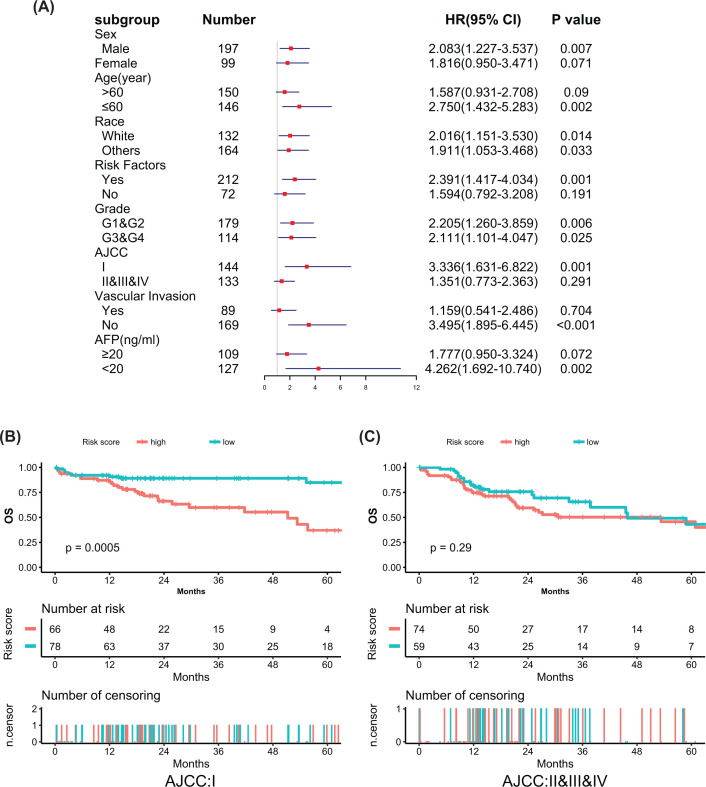
Stratification analysis of the predictive model (**A**) Univariate COX regression analysis of the predictive model in different subgroups stratified by important clinical variables. (**B,C**) Kaplan–Meier survival analysis of the predictive model in subsets of different AJCC stage patients with HCC (log-rank test). Other: Asian and black patients. Risk factors: hepatitis B, hepatitis C, hemochromatosis, cirrhosis, alcohol consumption, non-alcoholic fatty liver disease or α-1 antitrypsin deficiency.

Stratification analysis was performed using important clinical variables to further estimate the influence of the predictive model in the prognosis of HCC. The results showed that patients with high-risk scores had shorter OS in some HCC subgroups (Figure 7, Additional file 4: Supplementary Figure S2), including male patients (2.083 [95% CI, 1.227–3.537], *P*=0.007), patients ≤ 60 years (2.750 [95% CI, 1.432–5.283], *P*=0.002), patients with risk factors (2.391 [95% CI, 1.417–4.034], *P*=0.001), AJCC stage I patients (3.336 [95% CI, 1.631–6.822], *P*=0.001), patients without vascular invasion (3.495 [95% CI, 1.895–6.445], *P*<0.001) and patients with low AFP levels (4.262 [95% CI, 1.692–10.740], *P*=0.002). The race and histological grade of patients did not influence the performance of the model.

### Validation of the predictive model

A validation group was constructed by randomly selecting 148 HCC samples in the TCGA dataset, and ROC curve and survival analyses were used to further validate the prognosis predictive model. The results confirm that our predictive model is effective in the prognosis of HCC (Additional file 5: Supplementary Figure S3).

## Discussion

DNA methylation is a common mechanism of epigenetic regulation in eukaryotic organisms ranging from fungi to mammals [35]. Increased methylation of tumor-associated genes is an early event in many tumors [36,37]. HCC is one of the most common solid tumors; its incidence is increasing, and it has high mortality rates worldwide. Therefore, it is urgent to identify reliable prognostic markers and construct a robust risk model to predict HCC outcomes. In this study, we developed an effective DNA methylation-based prognosis predictive model.

To screen genes with different levels of methylation between HCC and non-tumor tissues, we first analyzed the DNA methylation data in multiple independent datasets to significantly enhance the reliability of our results. GO enrichment analysis and KEGG pathway enrichment analysis were performed to investigate the biological roles of CDMGs. As shown in Figure 2, CDMGs were clearly enriched in pathways involved in transcriptional misregulation in cancer and in Rap1 signaling. The important roles of these two pathways in the development and progression of HCC have been reported in many studies [38–41]. Therefore, bioinformatics analysis could enhance our understanding of the molecular and pathological mechanisms underlying HCC.

Furthermore, we identified four SR-CDMGs (*HOXA10, CDKL2, HOXB4* and *WDR69*) that were significantly associated with the survival of HCC patients. It has been reported that the homeobox (HOX) transcription factors play critical roles in many physiological and pathological processes [42,43]. Previous studies have reported that *HOXA10* contributes to tumorigenesis and progression in HCC [44–46]. In addition, it has been reported that the combination of *HOXA9* and *HOXA10* promoter CpG islands could predict the survival of breast cancer patients [47]. *HOXB4* has been reported to contribute to tumorigenesis in many cancers [48–51]. Moreover, *HOXB4* methylation could be a potential marker in human cervical cancer [52] and in extrahepatic cholangiocarcinoma [49]. Hyper-methylation of the *CDKL2* promoter has been suggested to play an important role in hepatocarcinogenesis and to be a potentially valuable biomarker for HCC [53,54].

*WDR69* is a member of the WD-repeat domain (WDR) family. Although its association with cancer has not been reported, an increasing number of members of the WDR family have been identified as involved in tumorigenesis and cancer progression, including (i) *WDR34*, which may be used as a prognosis predictor in breast cancer and may provide a novel target for the treatment of breast cancer [55], (ii) *WDR54*, which serves as an oncogene in colorectal cancer and may be a potential prognostic marker and therapeutic target [56], (iii) *WDR1*, which plays a predictive role in nodal metastasis for primary breast cancer [57], and (iv) *WDR34*, which inhibits oral squamous cell carcinoma progression [58]. In the present study, *WDR69* methylation was significantly negatively correlated with the survival of HCC patients. Furthermore, after analyzed the clinical and pathological features of HCC patients in the TCGA dataset, the results showed that the *WDR69* methylation level was higher in HCC tumor tissues with high histological grade, AJCC stage, and AFP levels (Additional file 6: Supplementary Figure S4). Therefore, the molecular functions of *WDR69* in HCC deserve to be further exploited. In summary, the potential molecular biological mechanism of the SR-CDMGs *HOXA10, CDKL2, HOXB4* and *WDR69* further supports the reliability of our results.

A previous study reported that altered DNA methylation patterns could be among the first detectable neoplastic changes associated with tumorigenesis [36,37]. DNA methylation alteration is frequently observed in HCC and is known to play important roles in carcinogenesis and diagnosis. Therefore, many studies have focused on DNA methylation in HCC, and several HCC methylation-based models have been proposed.

In terms of diagnostic prediction models, Cheng et al. identified six HCC-specific hyper-methylated sites (*NEBL* [cg23565942], *FAM55C* [cg21908638, cg11223367 and cg03509671], *GALNT3* [cg05569109], and *DSE* [cg11481534]) as potential diagnostic biomarkers, and the combination of these six sites showed a high sensitivity and a high specificity for the diagnosis of HCC [59]. Kisiel et al. identified a methylated DNA marker panel (*HOXA1, EMX1, AK055957, ECE1, PFKP* and *CLEC11A*), which was proven to accurately detect HCC by plasma tests [60]. Zhao et al. identified three DMGs, i.e., *ZNF300, SLC22A20* and *SHISA7*, which are potential markers for the early detection of HCC [61]. Zhong et al. reported that a panel of promoter methylations in *RNA5SP38, IL21* and *SDC4P* can serve in the diagnosis of HCC [62]. The study by Wu et al. supported the idea that methylation markers (cg10272601 in *WNK2*, cg12680131 in *TPO* and cg22511877 in *MYT1L*) in white blood cell DNA can serve as biomarkers of HCC susceptibility [63].

In terms of diagnostic and prognostic prediction model, Xu et al. successfully constructed a diagnostic prediction model using a 10-methylation marker panel (*BMPR1A, PSD, ARHGAP25, KLF3, PLAC8, ATXN1*, Chr 6:170, Chr 6:3, *ATAD2* and Chr 8:20) and a prognostic prediction model with an 8-methylation marker panel (*SH3PXD2A, C11orf9, PPFIA1*, Chr 17:78, *SERPINB5, NOTCH3, GRHL2* and *TMEM8B*) after screening for circulating tumor DNA methylation markers in HCC [64]. However, this study was limited by a relatively short follow-up period. Long et al. constructed prognostic and diagnostic models consisting of two DNA methylation-driven genes (*SPP1* and *LCAT*) [65] and Lu et al. showed that a combination of multiple methylation markers (*APC, COX2, RASSF1A* and miR-203) could accurately identify hepatitis B virus (HBV)-related HCC from patient plasma samples and exhibited diagnostic and prognostic potential [66], but the inclusion and exclusion criteria of HCC patients were not reported in these two papers.

In terms of prognostic prediction model, Li et al. developed a model to predict prognosis for HCC patients based on six methylation-driven genes (*SNHG6, S100P, DCDC2, LIME1, FMO3* and *GPR171*) [67]. However, again, the inclusion and exclusion criteria of HCC patients were not reported. Dong et al. constructed a DNA methylation-based survival prediction model for HCC based on 134 methylation sites [68]. However, due to the high number of methylated markers, this model is not convenient for clinical application. Qiu et al. reported that a three-CpG island signature (*SCAND3, SGIP1* and *PI3*) is useful in predicting recurrence for patients with early-stage HBV-related HCC [69]. However, it needs to be confirmed that it can be applied in patients with advanced-stage disease.

In our study, after excluding patients with detectable residual tumors (R1 and R2) and patients who received adjuvant treatment (including radiation therapy, chemotherapy and embolization treatment), we identified four SR-CDMGs and constructed a composite predictive model for HCC patients. The Kaplan–Meier curve showed that the OS were significantly different between different risk groups of HCC patients. Moreover, by Cox multivariate analysis and PSM analysis, we identified the risk score as an independent prognostic factor for poor prognosis, even after adjusting for important clinical variables. Furthermore, stratified analysis results showed that our model performed well in predicting the outcome of some HCC subgroups (mainly including male patients, patients aged ≤ 60 years, AJCC stage I patients, patients without vascular invasion and patients with low AFP levels). Besides, we constructed a validation group by randomly selecting 148 HCC samples in the TCGA dataset; the results of ROC curve analysis and survival analysis further validated our predictive model. In addition, because this model only uses a small number of prognostic markers, it is practical and easy to use for patient assessment and therapeutic decision making. In summary, we developed a relatively objective and effective DNA methylation-based predictive model that could provide more accurate and useful information in the prognosis of HCC.

In the future, we plan to further investigate the roles of these four methylated markers in HCC carcinogenesis. In particular, the molecular functions and mechanisms of *WDR69* in HCC deserve to be further exploited. Finally, this DNA methylation-based prognosis predictive model needs to be further validated.

## Conclusion

We identified four methylated biomarkers that are significantly associated with HCC survival, which might serve as potential therapeutic targets for HCC. Our DNA methylation-based prognosis predictive model is effective and reliable in the prediction of prognosis in HCC, which could be useful for patient stratification in clinical practice, facilitate clinical therapeutic decision-making and therefore improve the prognosis for patients with HCC.

## Supplementary Material

Supplementary Figures S1-S4

Supplementary Tables S1-S2

## Data Availability

The data included in the current study are available in the GEO database (https://www.ncbi.nlm.nih.gov/geo) and the TCGA database (https://cancergenome.nih.gov/).

## Abbreviations

AFP: α-fetoprotein
AJCC: American Joint Committee on Cancer
CDMG: common differentially methylated gene
DMP: differentially methylated probe
GO: Gene Ontology
HBV: hepatitis B virus
HCC: hepatocellular carcinoma
KEGG: Kyoto Encyclopedia of Genes and Genomes
LASSO: least absolute shrinkage and selection operator
OS: overall survival
PPI: protein–protein interaction
PSM: propensity score-matched
ROC: receiver operating characteristic
SR-CDMG: survival-related CDMG
TCGA: the Cancer Genome Atlas
WDR: WD-repeat domain
