# Supplementary material for: A novel DNA methylation-based model that effectively predicts prognosis in hepatocellular carcinoma
Source: Biosci Rep. 2021 Mar 10;41(3):BSR20203945. doi: 10.1042/BSR20203945 (PMC7955104; doi:10.1042/BSR20203945)
Supplement: Supplementary Figures S1-S4 [file BSR-2020-3945_supp.pdf]

Fig.S1

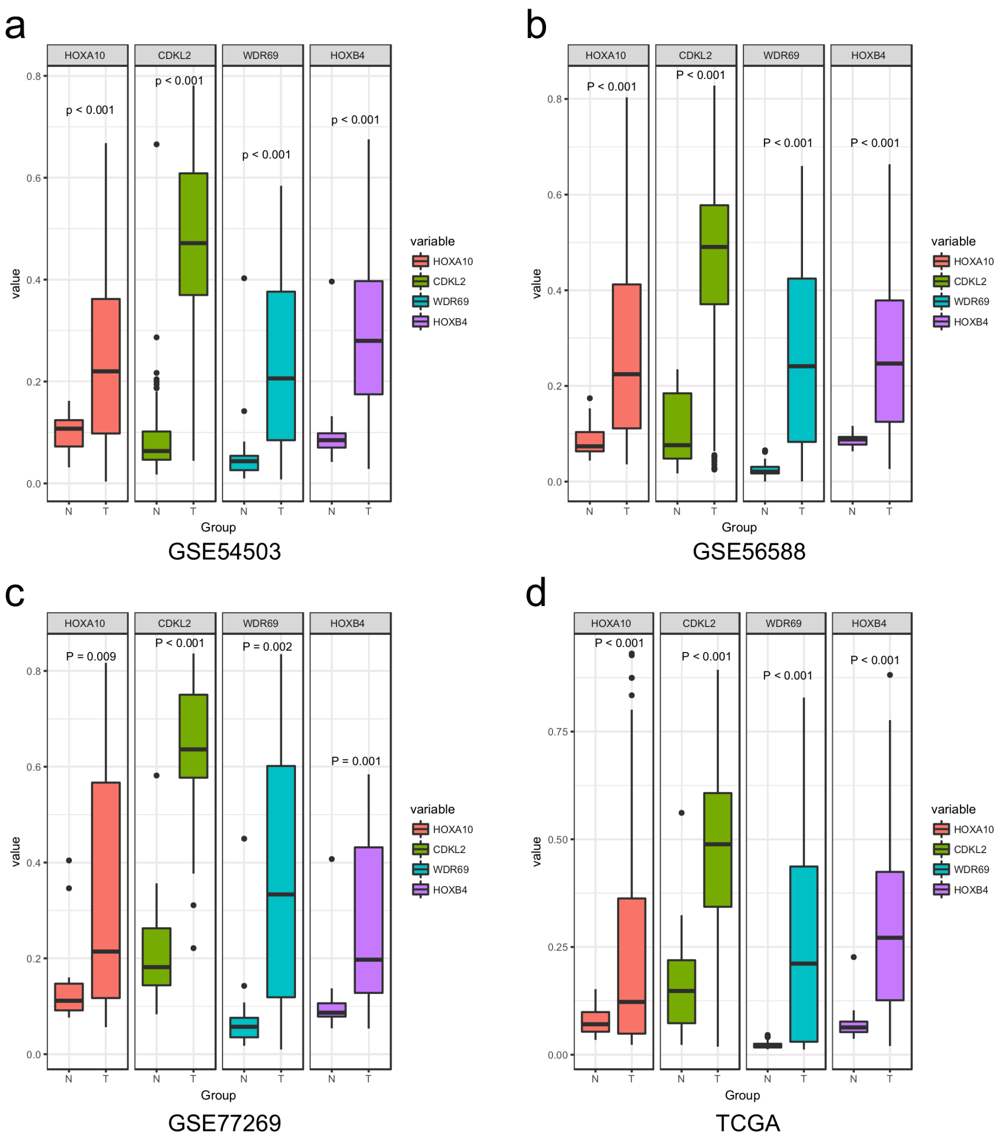

Supplementary material 3: Figure S1. Comparison of methylation level of the four SR-CDMGs between HCC and adjacent non-cancerous tissues.
(a) Methylation level of the four SR-CDMGs between T and N in GSE54503. (b) Methylation level of the four SR-CDMGs between T and N in GSE56588. (c) Methylation level of the four SR-CDMGs between T and N in GSE77269. (d) Methylation level of the four SR-CDMGs between T and N in TCGA. N, normal samples. T, tumour samples.

Fig.S2

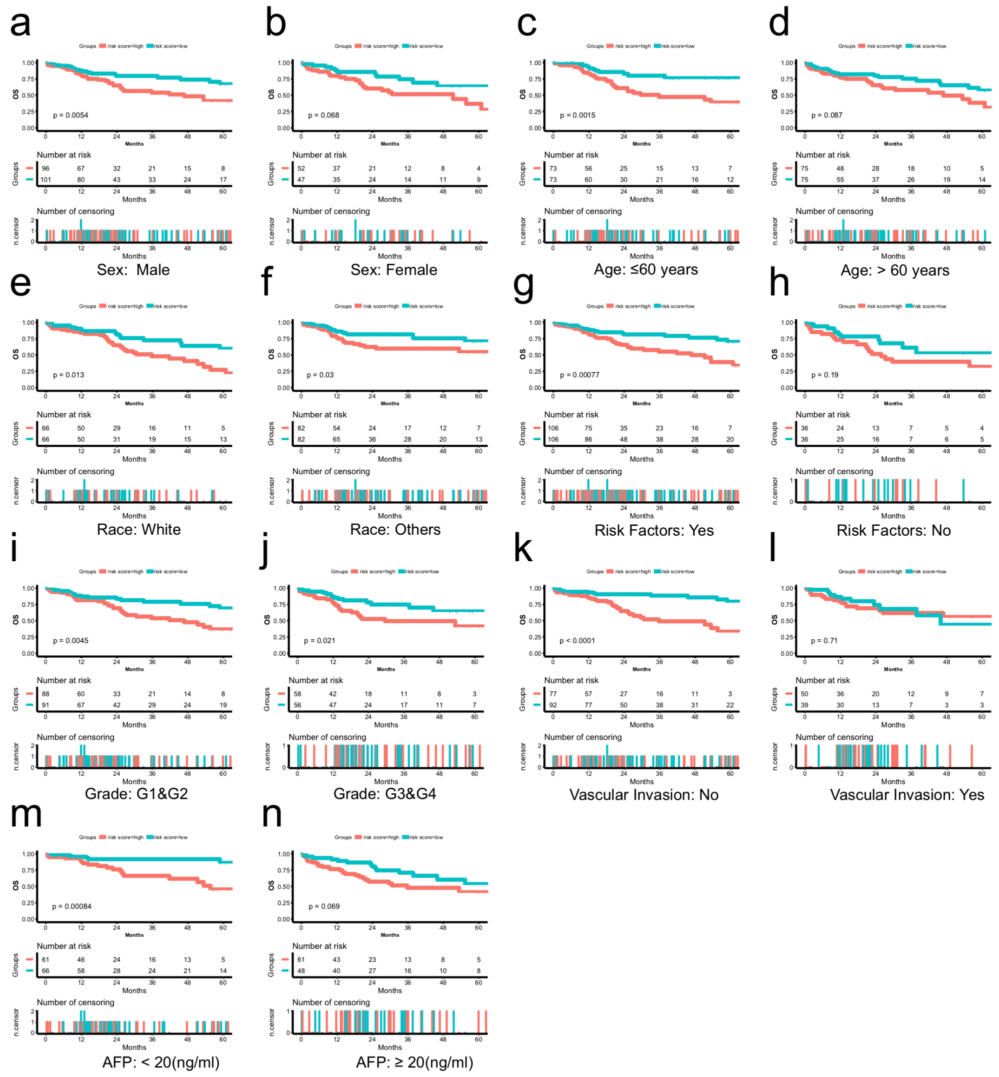

Supplementary material 4: Figure S2. Kaplan-Meier survival analysis of the predictive model in different subgroups of HCC patients. (a, b) sex, (c, d) age, (e, f) race, (g, h) risk factors, (i, j) histological grade, (k, l) vascular invasion, and (m, n) AFP level. P values were calculated with the log-rank test. Other: including Asian and black. Risk factors: representing hepatitis B, hepatitis C, hemochromatosis, cirrhosis, alcohol consumption, non-alcoholic fatty liver disease or alpha-1 antitrypsin deficiency.

Figure S3

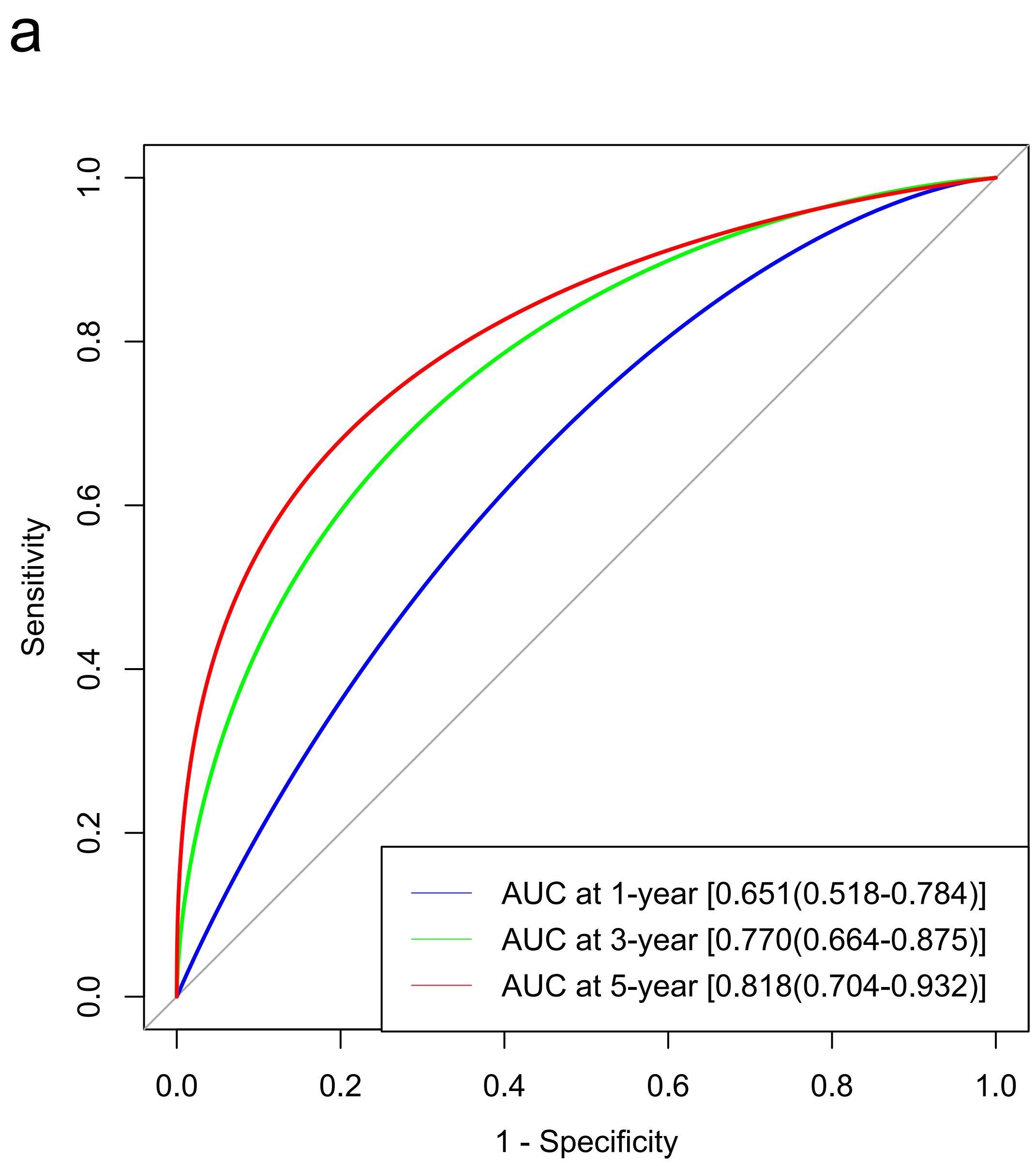

a
Sensitivity
1 - Specificity
0.0
0.2
0.4
0.6
0.8
1.0
AUC at 1-year [0.651(0.518-0.784)]
AUC at 3-year [0.770(0.664-0.875)]
AUC at 5-year [0.818(0.704-0.932)]

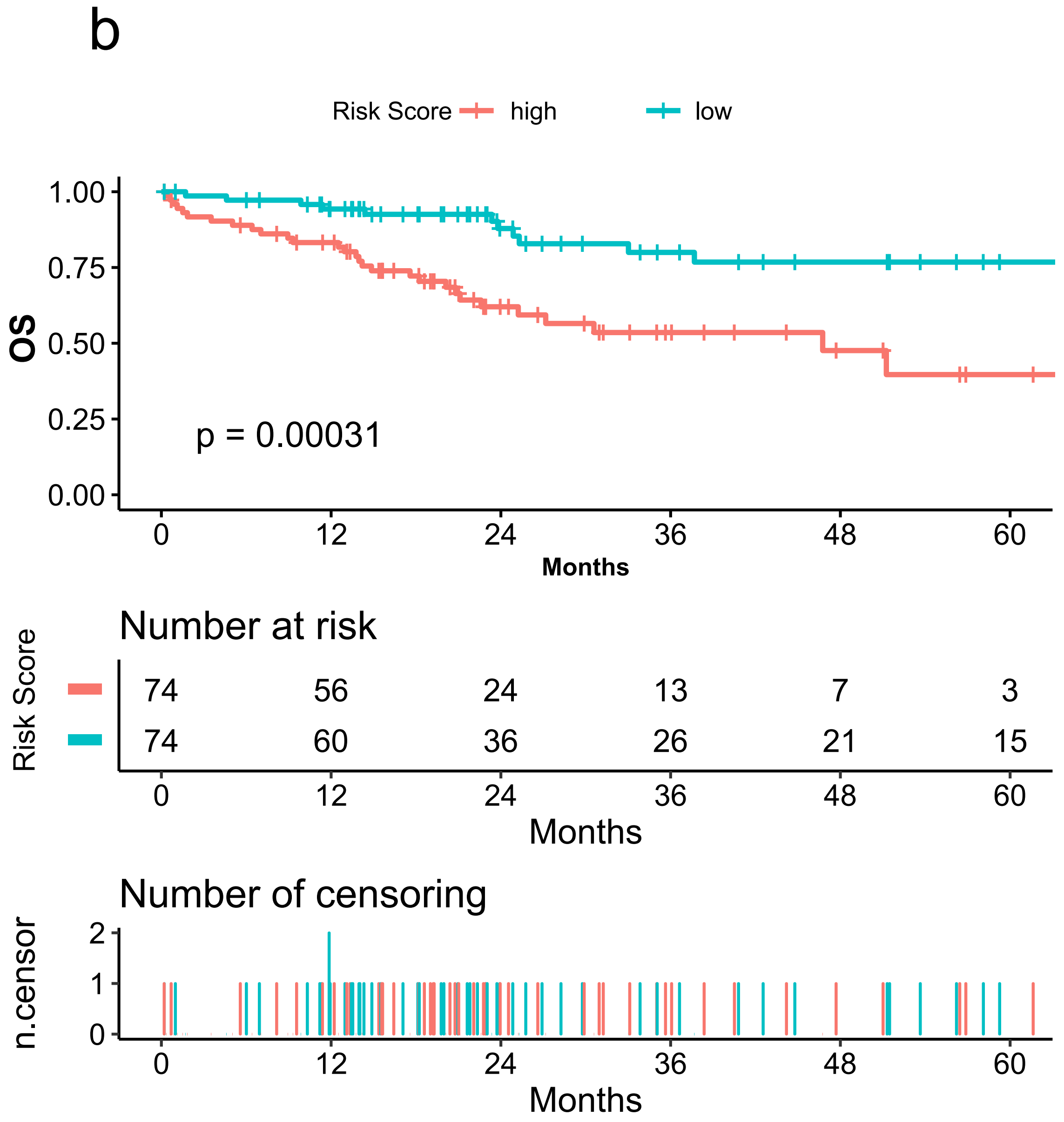

b
Risk Score
high
low
OS
1.00
0.75
0.50
0.25
0.00
p = 0.00031
Months
0
12
24
36
48
60
Number at risk
74 56 24 13 7 3
74 60 36 26 21 15
Number of censoring
n.censor
2
1
0

Additional file 5: Fig. S3 Validation of the predictive model

(a) AUC values for 1-, 3-, and 5-year survival of the predictive model. (b) Kaplan-Meier survival analysis of the predictive model.

Fig.S4

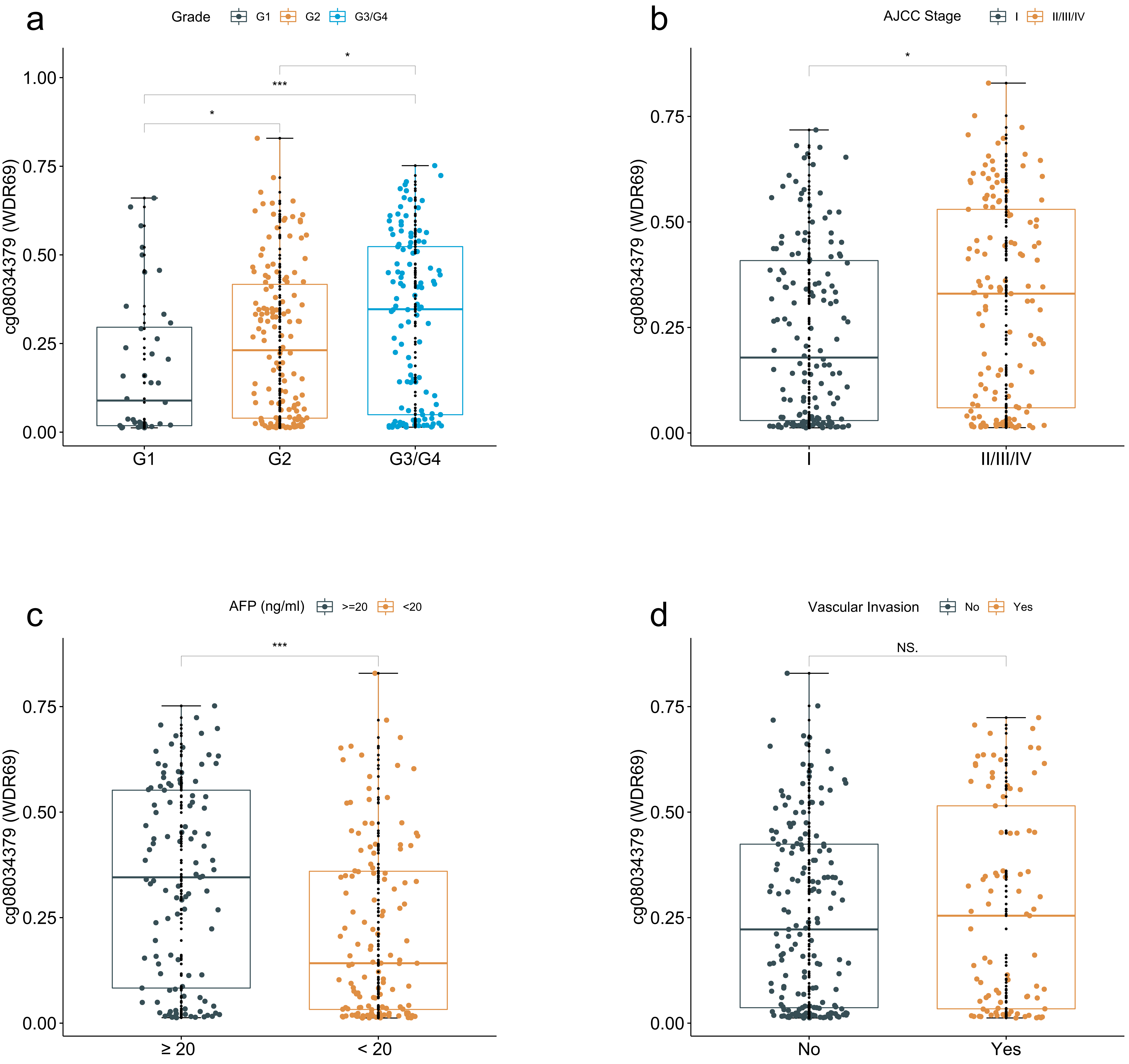
a
Grade
G1
G2
G3/G4
*
***
*
1.00
0.75
0.50
0.25
0.00
cg08034379 (WDR69)
G1
G2
G3/G4
b
AJCC Stage
I
II/III/IV
*
0.75
0.50
0.25
0.00
cg08034379 (WDR69)
I
II/III/IV
c
AFP (ng/ml)
>=20
<20
***
0.75
0.50
0.25
0.00
cg08034379 (WDR69)
≥ 20
< 20
d
Vascular Invasion
No
Yes
NS.
0.75
0.50
0.25
0.00
cg08034379 (WDR69)
No
Yes

Additional file 6: Fig. S4 *WDR69* methylation level was higher in HCC tumor tissues with high histological grade (a), AJCC stage (b), and AFP levels (c), but no significant difference in vascular invasion (d).

Additional file 7: Certificate of language modification
